# Electrophysiological Indices of Audiovisual Speech Perception in the Broader Autism Phenotype

**DOI:** 10.3390/brainsci7060060

**Published:** 2017-06-02

**Authors:** Julia Irwin, Trey Avery, Jacqueline Turcios, Lawrence Brancazio, Barbara Cook, Nicole Landi

**Affiliations:** 1 Haskins Laboratories, New Haven, CT 06511, USA; trey.avery@yale.edu (T.A.); jacqueline.turcios@yale.edu (J.T.); brancaziol1@southernct.edu (L.B.); 2 Department of Psychology, Southern Connecticut State University, New Haven, CT 06515, USA; 3 Department of Communication Disorders, Southern Connecticut State University, New Haven, CT 06515, USA; cookb5@southernct.edu; 4 Psychological Sciences, University of Connecticut, Storrs, CT 06269, USA

**Keywords:** audiovisual speech perception, development, broader autism phenotype, ERP

## Abstract

When a speaker talks, the consequences of this can both be heard (audio) and seen (visual). A novel visual phonemic restoration task was used to assess behavioral discrimination and neural signatures (event-related potentials, or ERP) of audiovisual processing in typically developing children with a range of social and communicative skills assessed using the social responsiveness scale, a measure of traits associated with autism. An auditory oddball design presented two types of stimuli to the listener, a clear exemplar of an auditory consonant–vowel syllable /ba/ (the more frequently occurring standard stimulus), and a syllable in which the auditory cues for the consonant were substantially weakened, creating a stimulus which is more like /a/ (the infrequently presented deviant stimulus). All speech tokens were paired with a face producing /ba/ or a face with a pixelated mouth containing motion but no visual speech. In this paradigm, the visual /ba/ should cause the auditory /a/ to be perceived as /ba/, creating an attenuated oddball response; in contrast, a pixelated video (without articulatory information) should not have this effect. Behaviorally, participants showed visual phonemic restoration (reduced accuracy in detecting deviant /a/) in the presence of a speaking face. In addition, ERPs were observed in both an early time window (N100) and a later time window (P300) that were sensitive to speech context (/ba/ or /a/) and modulated by face context (speaking face with visible articulation or with pixelated mouth). Specifically, the oddball responses for the N100 and P300 were attenuated in the presence of a face producing /ba/ relative to a pixelated face, representing a possible neural correlate of the phonemic restoration effect. Notably, those individuals with more traits associated with autism (yet still in the non-clinical range) had smaller P300 responses overall, regardless of face context, suggesting generally reduced phonemic discrimination.

## 1. Introduction

When a speaker talks, the consequences of this can both be heard and seen. Visual information about speech has been shown to influence what listeners hear, both in noisy environments (known as visual gain) [1] and when the auditory portion of the speech signal can be clearly heard (mismatched audiovisual speech demonstrates a visual influence in clear listening conditions, known as the McGurk effect) [2]. This influence of visible speech on hearing has been demonstrated in infancy [3,4,5,6]; further, typical speech and language development is thought to take place in this audiovisual (AV) context, fostering native language acquisition [7,8,9,10,11].

Deficits in audiovisual processing have been reported in children with autism spectrum disorders (ASD). For example, Foxe et al. (2013) [12] examined perception of visible speech in the presence of auditory noise in a cross-sectional sample of children and adolescents with ASD. School-aged ASD children (5–12-year-olds) showed less visual gain (i.e., an increased ability to identify what is being said when visible articulatory information is available) in the context of noisy speech than did controls, but this pattern did not appear in the adolescents with ASD (13–15-year-olds). Irwin, Tornatore, Brancazio & Whalen (2011) [13] tested children with ASD on a set of audiovisual speech perception tasks, including an AV speech-in-noise (visual gain) and a McGurk-type task. Given the possibility that previous reports of deficits in AV speech perception were simply due to less looking at the face of the speaker (a hallmark of autism), simultaneous eye fixation patterns were recorded and trials where the participant did not fixate on the speaker’s face excluded. Even when fixated on the speaker’s face, children with ASD were less influenced by visible articulatory information than typically developing TD controls, in speechreading (visual-only), speech-in-noise and with audiovisual mismatched (McGurk) stimuli. Using a task that isolates the mouth of the speaker, Iarocci, Rombough, Yager, Weeks & Chua (2010) [14] also report that children and adolescents with ASD are poorer at identifying what is said from visual-only stimuli (speechreading) and less likely to report a visually influenced response in a McGurk-type task. In addition to these behavioral findings, a recent study utilizing event-related potentials (ERP) found that adults with an ASD do not show a late effect of congruency (e.g., speaking face and voice matching, or mismatching) in frontal and central–parietal areas exhibited by typical controls, indicating that adults with ASD are less sensitive to mismatching AV speech stimuli [15]. Taken together, these studies suggest that individuals with ASD may have difficulty using visual speech information during perception of a speaking face. Given that natural listening environments are often noisy (e.g., classrooms, cafeterias, playgrounds), the ability to make use of visual articulatory information on the speaker’s face to repair the intended message is critical. Any loss in this ability to repair can lead to cascading negative effects in social communication, already a primary deficit for individuals with an ASD.

In addition to the auditory perception deficits observed in young and school-aged children with ASD described above, infant siblings of those with an ASD (who are at greater risk of ASD themselves) [16,17] also appear to be less visually influenced by mismatched AV speech stimuli [18]. Further, a recent ERP study found that infant siblings of individuals diagnosed with ASD show a prolonged latency in later P400 components in responding to direct gaze during face processing [19]. These findings suggest that family members of individuals with ASD, who share a higher proportion of their genome than unrelated individuals, may also process faces [19], and, potentially, speaking faces differently than TD perceivers [18]. These findings, implicating atypical face and audiovisual processing in siblings of individuals with ASD, are consistent with the fact that ASD has a significant genetic component and are heritable [20]; for a review, see [21]. Indeed, first-degree relatives of individuals with an ASD (both parents and siblings) have been reported to present sub-threshold behaviors associated with an autism diagnosis, commonly referred to as the “Broader Autism Phenotype” (BAP; [22,23,24,25]). Thus, both perceivers with ASD and those who exhibit the broader autism phenotype may be less visually influenced by a speaking face. Existing work on the BAP, consistent with a polygenic model of ASD in which commonly occurring genetic variation contributes to the autism phenotype, suggests that there is a distribution of autism-like traits in the general population [26]. However, there have been no examinations that we are aware of which have investigated AV speech processing in typically developing children that vary on measures of these traits. Given that visual influence on heard speech is known to vary among individuals [27], better understanding of this variability and whether autism-like traits contribute to this variability is of interest.

While existing studies are suggestive of atypical response to AV speech in ASD and siblings of children with ASD, the paradigms used to date for studying AV speech may not be ideal for use in this population because they make additional processing demands beyond the audio and visual speech. Specifically, studies that use speech in noise and/or mismatched (or McGurk-type) AV tasks have potential limitations for young children and those with ASD or ASD-like traits [28]. The McGurk effect creates a percept that differs from either the visual or auditory signal alone because of conflict between the two modalities, which results in percepts that are identified as poorer exemplars of a category [29]. This approach may be particularly problematic for those with ASD or ASD-like traits because weaknesses in executive function can lead to difficulties in identification of ambiguous stimuli [18,30]. Additionally, studying AV speech perception using paradigms that utilize auditory noise is problematic because noise is generally disruptive for individuals with ASD in the perception of speech [31]. In order to examine visual influence on heard speech in children who exhibit “autism-like traits” (or the broader autism phenotype), we have developed a measure that involves neither noise nor auditory and visual category conflict that can serve as an alternative to assessing audiovisual speech processing (also see [32] for a related approach). This new paradigm, which we describe in detail in Irwin et al. (accepted), uses restoration of weakened auditory tokens with visual stimuli. Two types of stimuli are presented to the listener: clear exemplars of an auditory consonant–vowel syllable (in this case, /ba/), and syllables in which the auditory cues for the consonant are substantially weakened, creating a stimulus which is more /*a*/-like, from this point on referred to as /*a*/. The auditory stimuli are created by synthesizing speech based on a natural production of the syllable and systematically flattening the formant transitions to create the /*a*/. Video of the speaker’s face does not change (always producing /ba/), but the auditory stimuli (/ba/ or /*a*/) vary. Thus, when the /*a*/ stimulus is dubbed over the visual /ba/, a visual influence will result in effectively “restoring” the weakened auditory cues so that the stimulus is perceived as a /ba/, akin to a visual phonemic restoration effect [33,34,35].

In the current paper, we examine behavioral discrimination and neural signatures (using event related potentials (ERP)) of audiovisual processing using this novel visual phonemic restoration method in children with typical development with a range of social and communicative skills. Given the dearth of ERP studies on AV speech perception in typically developing children and those with ASD, we look at the relation between AV speech processing and behavioral performance on the social responsiveness scale, which measures “autism-like traits” (SRS-2) [36]. As in Irwin et al. (accepted) [37], we utilize an oddball paradigm to elicit ERP responses to infrequently occurring /*a*/s embedded within the more frequently occurring intact /ba/s. All speech tokens are paired with a face producing /ba/ or a face with a pixelated mouth containing motion but no visual speech. If the visual /ba/ causes the auditory /*a*/ to be perceived as /ba/, then the oddball response to this stimulus should be attenuated. In contrast, a pixelated video (without articulatory information) should not have this effect. Therefore, in behavioral measures we predicted lower accuracy (detection of the oddball) for /*a*/ with a visual /ba/ (AV) than with a pixelated video (PX). In ERP, as a baseline we expected both early discrimination responses (within the first 200 ms, here observed as an N100) and a later P300 effect, with larger amplitudes to the infrequent /*a*/. Critically, we predicted that phonemic restoration from the visual /ba/ would result in a reduction in the amplitude increase for /*a*/ in the AV condition relative to PX (consistent with behavioral predictions). Further, we predict that children with higher scores on the SRS-2 (indicating an increased number of “autism-like traits”) will be less likely to use visual speech to effectively restore the /*a*/ to a full /ba/. This will result in those children with behaviors associated with ASD more likely to detect the /*a*/ in the AV speech condition in comparison to children with low SRS-2 scores. Likewise, in ERP, the children with high SRS-2 scores should show less of a difference between the AV and PX conditions in discrimination responses to /*a*/, relative to children with low SRS-2 scores.

## 2. Materials and Methods

All data presented here were collected according to the ethical guidelines laid out by the Yale University Institutional Review Board (HIC# 0312026173, approved 22 December 2016). Written consent was obtained from participants’ primary caregivers and written assent from child participants. 

### 2.1. Participants

Participants were 34 typically developing monolingual American English-speaking children (17 females and 17 males, age range 6.0 to 12.25, mean age = 9.22 years, SD = 1.77 years), recruited from the greater New Haven community. All participants were right-handed, passed vision screenings with a Snellen eye chart (vision as good as or better than 20/20) and hearing screening using a portable audiometer (responded to 500 Hz, 1000 Hz, 2000 Hz, 4000 Hz tones in each ear). Parents reported no history of developmental delay or disorder. Reported race and ethnicity was 25 Caucasian participants, 7 African American participants and 2 Hispanic participants.

### 2.2. Audiovisual Stimuli and Experimental Paradigm 

**Figure 1:**
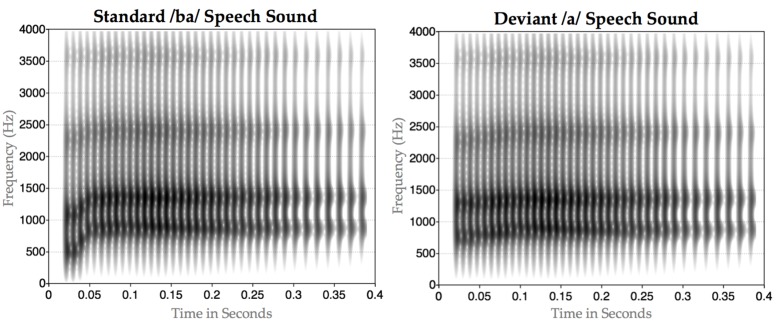
Spectrogram of /ba/ and /*a*/ synthesized auditory speech stimuli. First panel, top left, spectrogram of synthesized /ba/; Second panel, top right, edited synthesized /ba/ with reduced initial formants for the consonant, referred to as /*a*/.

The stimuli were created by videotaping and recording an adult male speaker of English producing the syllable /ba/. Using Praat [38], we extracted acoustic parameters for the token, including formant trajectories, amplitude contour, voicing and pitch contour. Critically, the token had rising formant transitions for F1, F2, and to a lesser extent F3, characteristic of /ba/. To create our /ba/ stimulus, we synthesized a new token of /ba/ based on these values. To create our /*a*/ stimulus we then modified the synthesis parameters: we changed the onset values for F1 and F2 to reduce the extent of the transitions and lengthened the transition durations for F1, F2 and F3, and then synthesized a new stimulus. For the /ba/, the transitions were 34 ms long and F1 rose from 500 Hz to 850 Hz; F2 rose from 1150 Hz to 1350 Hz; and F3 rose from 2300 Hz to 2400 Hz. For the /*a*/, the transitions were 70 ms long and F1 rose from 750 Hz to 850 Hz; F2 rose from 1300 Hz to 1350 Hz; and F3 rose from 2300 Hz to 2400 Hz (see Figure 1).

**Figure 2:**
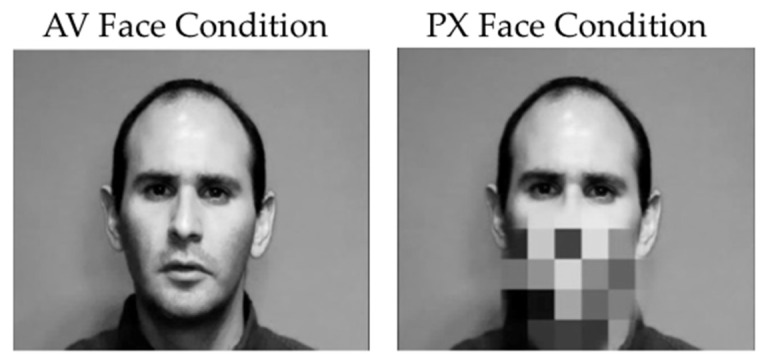
Image of audiovisual (AV) and pixelated (PX) face condition stimuli. Left panel audiovisual face condition, showing the visible articulation of the speaker; Right panel pixelated face condition, showing the speaker’s face, but obscuring the mouth.

To produce the AV stimuli, the /ba/ and /*a*/ synthesized auditory stimuli were dubbed onto video of the speaker producing /ba/, with the acoustic onsets synchronized with the visible articulation. A second condition was created in which the mouth portion of the video was pixelated so that the articulatory movement surrounding the mouth could not be perceived but general head movement was preserved (although variation in the pixelation indicated movement). As in the AV condition, the synthesized /ba/ and /*a*/ stimuli were dubbed onto the pixelated video (see Figure 2 below).

Instructions and a practice trial were presented prior to the start of the experiment. Within the full electroencephalography (EEG) session, the experiment was blocked into two face context conditions (AV and pixelated face, see Figure 2). Each face context block was 18 min and contained 200 trials lasting 2 s each. After each 50 trials, the participant was given a break and instructed to press the response button when they were ready to resume the experiment. First was the (AV) block (where the speaking face was fully visible) and second was the pixelated (or PX) block (where the area around the mouth was pixelated to obscure features of the mouth). This presentation order was intentional to ensure that the phonemic restoration effect was tested without exposure to the contrast of the /ba/ and /*a*/ auditory tokens, which should be clearly contrastive without the visible articulation from the mouth. An 80/20 oddball design was used for presentation of the speech stimuli, with /*a*/ serving as the infrequently occurring (or deviant stimulus) in both face contexts. Participants were played the deviant sound (/*a*/) before each block to remind them what they were listening for, and instructed to press the response button only after the presentation of that deviant stimulus and to otherwise remain as still as possible. Total experiment time was approximately 45 min depending on length of breaks and amount of EEG net rehydration between blocks.

### 2.3. EEG Data Collection

EEG data was collected with an Electrical Geodesics Inc. (EGI) netamps 300 high-impedance amplifier, using 128 Ag/AgCl electrodes embedded in soft sponges woven into a geodesic array. The EEG sensor nets were soaked for up to ten minutes prior to use in a warm potassium chloride solution (2 teaspoons of potassium chloride, 1 liter of water purified by reverse osmosis, and 3 ccs of Johnson & Johnson baby shampoo to remove oils from the scalp). The high-density hydrocel geodesic sensor nets and associated high-impedance amplifiers have been designed to accept impedance values ranging as high as 100 kΩ, which permits the sensor nets to be applied in under ten minutes and without scalp abrasion, recording paste, or gel (e.g., [39,40]). Impedance for all electrodes was kept below 40 kΩ throughout the experimental run (impedances were re-checked between blocks). Online recordings at each electrode used the vertex electrode as the reference and were later referenced to the average reference.

EEG was continuously recorded using Netstation 5.2 on a MacPro running OS X 10.11 while participants completed experimental tasks. Stimuli were presented using E-Prime version 2.0.8.90 (Psychology Software Tools, Inc., Sharpsburg, PA, USA) on a Dell Optiplex 755 (Intel Core 2 Duo at 2.53 GHz and 3.37 GB RAM) running Windows XP. Audio stimuli were presented from an audio speaker centered 85 cm above the participant connected to a Creative SB X-Fi audio card. Visual stimuli were presented at a visual angle of 23.62 degrees (video was 9.44 inches/24 cm wide and 7.87 inches/20 cm tall) on Dell 17 inch flat panel monitors 60 cm from the participant connected to an Nvidia GeForce GT 630 video card. Speech sounds were presented free field at 65 decibels, measured by a sound pressure meter.

### 2.4. ERP Data Processing

Initial processing was conducted using Netstation 4.5.7. EEG data were band-pass filtered at .3 to 30 Hz (Passband Gain: 99.0% [−0.1 dB], Stopband Gain: 1.0% [−40.0 dB], Rolloff: 2.00 Hz) and segmented by condition, 100 ms pre-stimulus to 800 ms post-stimulus. In order to balance the number of standards and deviants in the ERP analysis, only the standard before each deviant was included for analysis, resulting in 40 possible trials for each standard and deviant speech sound in each face context.

**Figure A1:**
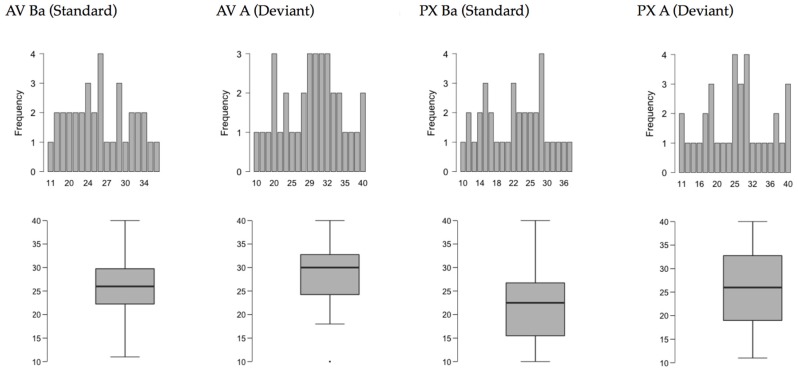
Frequency distribution of useable trials as a function of face context and speech stimulus.

Eye blinks and vertical eye movements were examined with electrodes located below and above the eyes (channels 8, 126, 25, 127). Horizontal eye movements were measured using channels 125 and 128, located at positions to the left and right of the eyes. Artifacts were automatically detected and manually verified for exclusion from additional analysis (bad channel >200 microvolts, eye blinks >140 microvolts and eye movement >55 microvolts). For every channel, 50% or greater bad segments was used as the criteria for marking the channel bad; for every segment, greater than 20 bad channels was used as a criterion for marking a segment bad. Participants with less than 25% of a possible 40 usable trials in any condition were excluded from analysis. The average usable trial count across all conditions was a mean of 26.21 (SD = 8.81) and each experiment had similar amounts of usable data, AV mean = 27.88 (SD 8.79) and PX mean = 24.41 (SD 8.83) (See Appendix Figure A1 for more detail on the frequency distribution of usable trials by condition.). Collapsing standards and deviants, there were similar quantities of usable trials in the grand average with a mean of 24.52 (SD = 8.46) standards and mean = 27.78 (SD = 9.15), deviants.

Bad channels (fluctuations over 200 μV) were spherical spline interpolated from nearby electrodes. Data were baseline-corrected using a 100 ms window prior to onset of all stimuli. Data were re-referenced from vertex recording to an average reference of all 128 channels. For ERP analysis, only standard /ba/ sounds before the deviant (/*a*/) were included.

**Figure 3:**
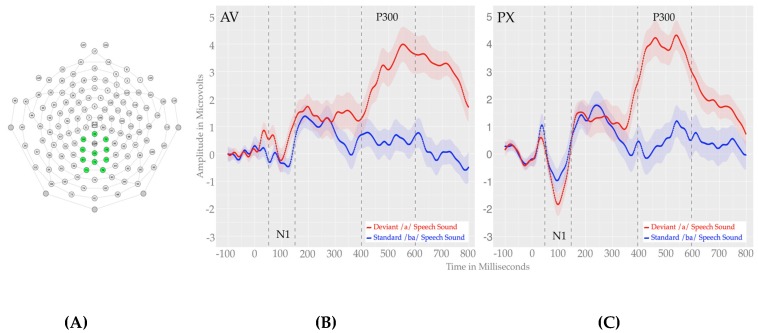
Waveform plots showing the N100 and P300. (**A**): electrode montage; (**B**) and (**C**): N100 and P300 response to standard /ba/ and deviant /*a*/ in the audiovisual (AV) condition; Right panel: N100 and P300 response to standard /ba/ and deviant /*a*/ in the pixelated video (PX) condition. Shading around waveforms represents the standard error from the mean.

All processed, artifact-free segments were averaged by condition producing a single event-related potential waveform for each condition for all participants and exported for plotting and statistical analysis in R. Visual inspection of our data for the PX condition revealed a clear negative peak around 100 milliseconds (the N100) and a large positive P300 component that began around 380 milliseconds and returned to baseline between 600 and 700 ms (see Figure 3). For the AV condition, similar components were clearly visible, though the N100 was visibly reduced for the AV condition and the P300 was larger and visibly flattened for the AV condition. Electrode montages and temporal windows were selected based on component identification in the PX condition as this represents a “baseline” speech contrast condition. For analysis, we defined the N100 as the most negative peak between 50 and 100 ms and the P300 to be the most positive peak between 400 and 600 ms following stimulus onset, both within a cluster of eleven central electrodes (see Figure 3). In addition, because of the large temporal extent of the P300 and visible slope differences in the early portion of the P300 window, we take a two-stage approach to our P300 analyses. First, we conducted an analysis on the full 400–600 ms window (chosen based on inspection of individual subject data in the PX condition to ensure capture of the P300) as well as early (400–500 ms) and late windows (500–600 ms). Initial identification of the N100 and P300 were based on both visual inspection and guidelines provided by previous ERP studies of speech sound discrimination [41]. To examine the ERP effects of speech stimulus (standard /ba/ vs. deviant /*a*/) as a function of face context (mouth visible (AV) vs. mouth pixelated (PX)), we ran separate 2 × 2 repeated measures ANOVAs with speech stimulus (standard /ba/ vs. deviant /*a*/) and face context (AV vs. PX) as within-subjects variables for each of our components of interest (N100, overall P300, early P300 and late P300). These analyses were conducted on average amplitudes that included a window of 25 ms around the peak within each window (5 ms on each side of the N1 and 12.5 ms on each side of the P300), identified using an adaptive mean function, which identifies individual windows for each participant to account for subtle differences in waveform morphology across participants.

### 2.5. Assessment Data

The Social Responsiveness Scale Second Edition (SRS-2) was completed by all participants’ primary caregivers as part of a larger study examining audiovisual integration in relation to autism spectrum disorder. The SRS-2 was designed to identify “autism-like traits”, which are separated into five treatment subscales. The subscales within the SRS-2 include: social awareness, social cognition, social communication, social motivation and restricted interests and repetitive behavior. The set of subscales are meant to identify behaviors that a child may have in any of the five areas for designing and evaluating treatment. The total score is utilized to indicate severity of social deficits to identify children with autism or social communication impairment, aligning with criteria from the Diagnostic and Statistical Manual of Mental Disorders, Fifth Edition (DSM-V). Children may be rated in three different categories: mild, moderate and severe. A higher overall score will mean greater severity and greater number of behaviors characteristic of an ASD [36].

## 3. Results

### 3.1. Parent Report on the Social Relatedness Scale (SRS-2) 

Participant scores ranged from 38 to 65 on the SRS-2, with a mean score of 45.58 (SD 5.69). A score of 59 or lower is considered “within normal limits”; a score of 60 to 65 is considered to be in the “mild range”; 66 to 75 is considered to be in the moderate range and a score of 76 is typically indicative of children who have autism. A score ranging from mild to severe is considered have clinical significance and warrants further evaluation for an autism spectrum disorder. In our sample, one child scored in the mild range and all other children scored within the normal range. However, four participants received scores that were in the 50–59 range and 24 received scores in the 40–49 range. 

### 3.2. Behavioral Response in our ERP Experiment

Participants pressed a button when they perceived a deviant /*a*/ in both AV and PX conditions. Responses were reported as a percentage of correct responses out of a possible 40 trials in each face context, that is, detection of the oddball */a/* stimulus. As expected, participants responded to the deviant oddball target stimuli more accurately in the PX condition mean = 84.48 (SD = 22.70) compared to the AV condition mean = 45.54 (SD = 40.69). A pairwise comparison of response accuracy revealed a significant difference between AV and PX conditions *t*(27) = −5.696, *p* < 0.001, *d* = −1.076.

### 3.3. ERP Data

#### 3.3.1. N100

**Figure 4:**
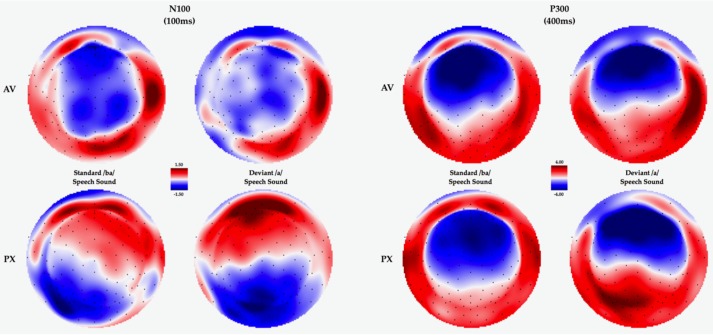
Topomaps for the N100 and P300 effects by condition.

For the N100 time window, we observed a main effect of face context, F(1, 33) = 6.199, *p* = 0.018, *η*^2^ = 0.158. The main effect of speech stimulus was not significant, F(1, 33) = 0.010, *p* = 0.920, *η*^2^ = 0.000. We also observed a marginal interaction face context and speech stimulus F(1, 33) = 3.97, *p* = 0.055, *η*^2^ = 0.107, suggesting differential modulation of phoneme perception as a function of face context. Because our predictions implicate specific differences for the phoneme perception as a function of face context, we conducted planned comparisons (two-tailed *t*-tests) comparing N100 response within the /*a*/ condition and within the /ba/ condition, across the two face context conditions. These comparisons revealed more a negative N100 response in the PX condition compared to the AV condition for the deviant /*a*/, *t*(33) 3.485, *p* = 0.001, *d* = 0.598 but not for the standard /ba/, *t*(33) 0.801, *p* = 0.429, *d* = 0.137 (see Figure 3 and Figure 4) . This effect suggests attenuation of response to the deviant /*a*/ in the presence of the visual /ba/, indicating a phonemic restoration effect. 

#### 3.3.2. P300

For the full P300 time window (400–600 ms), we observed a main effect of speech stimulus, F(1, 33) = 27.273, *p* < 0.001, *η*^2^ = 0.505, with a more positive ERP response to deviant /*a*/s relative to standard /ba/s. The main effect of face context was not significant F(1, 33) = 0.576, *p* = 0.453, *η*^2^ = 0.017 and there was no interaction between face context and speech stimulus F(1, 33) = 1.324, *p* = 0.258, *η*^2^ = 0.039.

In the early P300 time window (400–500 ms), we observed a significant main effect of speech stimulus F(1, 33) = 34.956, *p* < 0.001, *η*^2^ = 0.514 with a more positive ERP response to deviant /*a*/s relative to standard /ba/s. The main effect of face context was not significant F(1, 33) = 1.645, *p* = 0.209, *η*^2^ = 0.047. We also observed a marginally significant speech stimulus by face context interaction F(1, 33) = 3.089, *p* = 0.088, *η*^2^ = 0.086. Given our prediction of a reduced P300 effect in the presence of a face speaking /ba/, we ran a planned contrast (two-tailed *t*-test) on the difference waveform (/*a*/ vs. /ba/) between the two face contexts. This analysis revealed a marginally larger speech stimulus effect in the PX condition compared to the AV condition PX versus AV *t*(33) −1.775, *p* = 0.087, *d* = −0.335. This trend suggests some attenuation of the early P300 in the presence of the visual /ba/, indicating phonemic restoration. 

In the late P300 time window (500–600 ms), we observed a significant main effect of speech stimulus F(1, 33) = 27.273, *p* < 0.001, *η*^2^ = 0.452, with larger amplitudes for deviant /*a*/s relative to standard /ba/s. The main effect of face context was not significant, F(1, 33) = 0.604, *p* = 0.443, *η*^2^ = 0.018 and there was no interaction between face context and speech stimulus F(1, 33) = 0.001, *p* = 0.974, *η*^2^ = 0.000. P300 effects are shown in Figure 3 and Figure 4.

#### 3.3.3. Correlations Between ERP Responses and SRS-2 Scores

**Figure 5:**
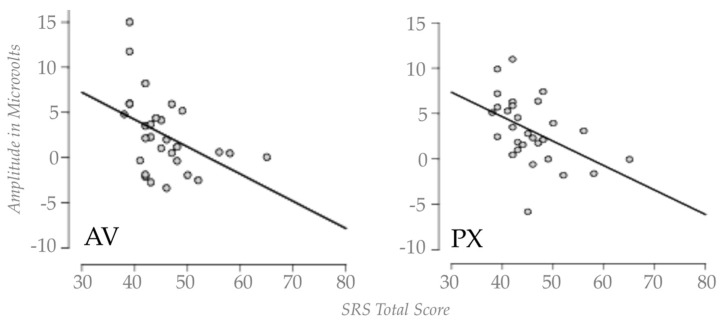
Scatter plots showing the correlation between the late P300 effect and the social responsiveness scale (SRS) total score in the AV and PX conditions.

Pearson’s correlations were computed to evaluate the relationship between task accuracy of each ERP component effect (subtraction of the deviant /*a*/ amplitude from the standard /ba/ amplitude for the N100, the, early P300, and late P300 in both the AV and PX face contexts) and SRS-2 total scores (SRS-2 total scores consist of a sum of the five sub scales; exploratory sub-scale correlations can be found in Appendix Table A1). The only significant correlation we observed was a negative correlation between the SRS-2 score and the late P300 and (that is, a smaller P300 effect was associated with higher scores on the SRS-2) for both the AV (*r* = −0.433, *n* = 34, *p* = 0.019) and PX (*r* = −0.458, *n* = 34, *p* = 0.012) face contexts (scatter plots of this effect are shown in Figure 5). Given three correlations for each face context condition, we applied a Holm–Bonferroni correction with a threshold of 0.05 [42] to each set of three correlations to correct for multiple comparisons; after correction our pattern of findings remained the same, with the correlation between both the AV and PX late P300 and SRS-2 scores remaining significant, with an adjusted *p* = 0.024. Thus, children with more autism-like symptoms had smaller P300 effects overall, regardless of face context.

## 4. Discussion

As predicted, our behavioral measure of discrimination (accuracy) indicated that children found the deviant /*a*/ more difficult to discriminate in the context of a visual articulation of /ba/, suggesting a visual phonemic restoration effect for the /*a*/ token in the AV condition.

With respect to our ERP findings, we observed both early N100, and later P300 effects, which were differentially modulated by speech stimulus or face context. We also observed a marginal speech stimulus by face context interaction for both the N100 and Early P300. For this early N100 effect, the response overall was more negative and had a sharper peak in the PX condition relative to the AV, consistent with previous findings [43] suggesting reduced amplitudes and ERP effects during audiovisual speech relative to speech with no visible articulation. Additionally, we observed a marginal interaction for this component which was driven by the more negative response to the deviant /*a*/ in the PX condition relative to the deviant/*a*/ in the AV condition, but no difference between the standard /ba/ in the PX relative to the AV condition. This marginal effect may reflect a reduction in early discrimination for the /ba/ versus /*a*/ contrast in the AV condition, consistent with our overall prediction of phonemic restoration for the AV condition.

For the P300 effect, we broke down our analysis into the overall effect (entire P300 window) and an early and late P300 based on our observation of differential slopes for the early aspect of the P300 as a function of face context, with the early P300 eliciting a faster rising and sharper peak in the PX condition relative to the AV condition. The overall analysis yielded a main effect of speech stimulus, with more positive amplitudes for deviants relative to standards (classic P300 response) but no modulation as a function of face context condition. In the early P300 window, we also observed a main effect of speech stimulus, and we observed a marginal interaction of face context and speech stimulus, such that larger early P300 effects were observed in the PX relative to the AV condition, consistent with predictions for phonemic restoration in the AV condition. For the later P300 window, we observed only a main effect of speech stimulus.

To explore whether the broader autism phenotype was associated with any of our ERP components of interest, we ran correlation analyses between SRS-2 scores and ERP component effects for the N100 and both the early and late P300 (based on a subtraction of the standard response from deviant response for both face contexts). The only significant correlation we observed was with the late P300 effect, for both the AV and PX conditions. This was a negative correlation, such that higher scores on the SRS-2 were associated with reduced late P300 response, regardless of face context. Thus, those individuals with more traits associated with autism (yet still in the non-clinical range) had smaller P300 responses overall, regardless of face context, suggesting generally reduced phonemic discrimination. In general, this finding is consistent with previous work with children with ASD which has found reduced P300 and mismatch negativity (MMN) effects in auditory discrimination tasks (e.g., P300) [44], MMN [45], and may suggest less attention to phonemic features, independent of visual speech.

It is worth noting that our findings in the present study with children differ somewhat from our previously reported findings using a similar paradigm with adults (Irwin et al. accepted). Namely, in that study we observed phonemic restoration effects in the overall P300 effect and in the mismatch negativity MMN response; here we observed marginal phonemic restoration effects in the early P300 effect and in the N100. Given the similar pattern of accuracy findings across the two studies, with reductions in deviant /*a*/ detection in the context of a visual articulation of /ba/ (though children were less accurate overall), these differences may reflect immature or more distributed neural systems for AV speech processing in children, which may produce less parity in the relationship between behavioral performance and neural response under AV speech processing conditions.

## 5. Conclusions

Behavioral effects of phonemic restoration, evidenced by reduced detection of infrequently presented /*a*/s among frequently presented /ba/s in the presence of a speaking face that produced a visual /ba/ were observed in school-aged children. We also observed ERPs in both an early time window (N100) and a later time window (P300) that were sensitive to phonemic perception and modulated by face context (speaking face vs. pixelated face). Specifically, the N100 response was modulated by face context such that more negative waveforms to the deviant /*a*/ were observed in the pixelated condition, reflecting an early neural correlate of the phonemic restoration effect. Further, the early P300 was sensitive to both the speech contrast (/ba/ vs. /*a*/) and face context, revealing a marginally larger early P300 response for the PX condition, representing a second possible neural correlate of the phonemic restoration effect. Finally, the SRS, which measures “autism-like traits”, was correlated with the late P300 response, suggesting reduced sensitivity to phonemic features in general for children with more behaviors associated with autism. 

## Appendix

**Table A1:** Correlations between each of the SRS subscale scores and between the late P300 effect for the PX and AV conditions and each of the SRS-2 subscale scores.

Pearson Correlations												
		SRS Total	SRS Awr	SRS Cog	SRS Com	SRS Mot	SRS RRB	SRS SCI	AV P300 Effect	Holm–Bon Correct p	PX P300 Effect	Holm–Bon Correct p
SRS Total	Pearson’s r	—	0.794	0.827	0.955	0.778	0.733	0.989	−0.433		−0.458	
	*p*-value	—	<0.001	<0.001	<0.001	<0.001	<0.001	<0.001	0.019	0.114	0.012	0.060
SRS Awr	Pearson’s r		—	0.522	0.711	0.383	0.660	0.773	−0.376		−0.320	
	*p*-value		—	0.004	<0.001	0.041	<0.001	<0.001	0.044	0.145	0.091	0.182
SRS Cog	Pearson’s r			—	0.758	0.614	0.607	0.829	−0.407		−0.370	
	*p*-value			—	< .001	< .001	< .001	< .001	0.029	0.145	0.048	0.144
SRS Com	Pearson’s r				—	0.757	0.589	0.965	−0.367		−0.497	
	*p*-value				—	<0.001	<0.001	<0.001	0.050	0.145	0.006	0.042
SRS Mot	Pearson’s r					—	0.411	0.811	−0.251		−0.392	
	*p*-value					—	0.027	<0.001	0.188	0.188	0.035	0.140
SRS RRB	Pearson’s r						—	0.641	−0.450		−0.084	
	*p*-value						—	<0.001	0.014	0.098	0.664	0.664
SRS SCI	Pearson’s r							—	−0.402		−0.484	
	*p*-value							—	0.030	0.145	0.008	0.048
AV P300 Effect	Pearson’s r								—		0.284	
	*p*-value								—		0.104	
PX P300 Effect	Pearson’s r										—	
	*p*-value										—	

Awr = Social Awareness; Cog = Social Cognition; Com = Social Communication; Mot = Social Motivation; SCI = Sum of the four previous subscales; RRB = Restricted Interests and Repetitive Behavior; Holm–Bon Correct = Holm–Bonferroni correction.
